# Qualitative Study on Antimicrobial Usage and Resistance in the Dairy Chain: A Situation Analysis and Solutions by Stakeholders from Punjab, India

**DOI:** 10.3390/antibiotics11091229

**Published:** 2022-09-09

**Authors:** Deepthi Vijay, Jasbir Singh Bedi, Pankaj Dhaka, Randhir Singh, Jaswinder Singh, Anil Kumar Arora, Jatinder Paul Singh Gill

**Affiliations:** 1 Centre for One Health, College of Veterinary Science, Guru Angad Dev Veterinary and Animal Sciences University, Ludhiana 141004, India; 2 Department of Veterinary and Animal Husbandry Extension Education, College of Veterinary Science, Guru Angad Dev Veterinary and Animal Sciences University, Ludhiana 141004, India; 3 Department of Veterinary Microbiology, College of Veterinary Science, Guru Angad Dev Veterinary and Animal Sciences University, Ludhiana 141004, India

**Keywords:** antimicrobial resistance, dairy chain, qualitative analysis, stewardship, veterinary services

## Abstract

The rising prevalence of antimicrobial resistance in animal foods and injudicious antibiotic use in the dairy sector pose significant threats to public health. Focus group discussions (FGDs) and key informant interviews (KIIs) were conducted with a strategic sample of four stakeholder groups (114 participants) associated with antibiotic usage in the dairy sector of Punjab. The FGDs were conducted among veterinarians (*n* = 56), para-veterinarians (*n* = 28), and KIIs were conducted among chemists (*n* = 18) and dairy quality managers (*n* = 12) during 2020–2021. FGDs and qualitative interviews of various stakeholders depict existing risk practices in the fields that may promote antimicrobial resistance. The present study revealed that widely prevalent quackery (treatment practices carried out by unauthorized persons without any recognized diploma/degree) and self-treatment by farmers, over-the-counter availability of antibiotics, low veterinarian per animal ratio, and lack of awareness among the society about the potential public health effects of antimicrobial resistance were the main risk factors for injudicious antibiotic use in the dairy sector. The present study involved a comprehensive approach targeting the stakeholders in the dairy sector and their reflections on judicious antimicrobial usage and antimicrobial resistance adapted to the dairy farming of Punjab. There is an urgent need for the advocation of policies that consider the specific challenges faced by the dairy sector to simultaneously improve access to veterinary services as well as strengthen antibiotic stewardship.

## 1. Introduction

Antimicrobial resistance (AMR) is one of the greatest public health challenges and is considered one of the next possible pandemics of the 21st century [1]. By 2030, the livestock industry is projected to account for 70% of the total antimicrobial usage (AMU) globally, and the AMU in the animal husbandry sector of India is predicted to double by this time period [2]. The inappropriate use of antimicrobials in food animals, especially in developing nations, is found to augment the transmission of antimicrobial resistant bacteria and antimicrobial resistant genes to humans through direct and indirect transmission mechanisms, such as direct contact with animals harboring AMR pathogens and their environments, through contaminated animal foods, such as milk, meat, and crops irrigated with livestock manure [3,4]. Thereby, the issue of AMR needs to be addressed in a ‘One Health’ framework and there is an urgent need to overcome interdisciplinary barriers and promote more cross-field research involving human and animal sectors [5].

India was ranked as one of the top 10 countries with the highest veterinary antimicrobial consumption in 2017 [6]. The emerging intensive farming practices of the country have been posited as the reasons for the large-scale use of antibiotics, making the country one of the hotspots of AMR. The possible factors for increasing AMR, especially in developing countries, including India, have increased, along with indiscriminate use of antibiotics in animal production, poor farm biosecurity practices, inadequate infection control practices in consort with the lack of compliance with regulatory frameworks [7,8,9].

Qualitative research approaches, such as focus group discussions (FGDs), are suitable for gaining an in-depth understanding of social issues [10]. They act as a bridging link between scientific research and people’s perceptions, knowledge, attitude, and actions [11], and are critical for decision-making on sensitive social issues by accessing the thoughts and feelings of the subjects [12]. In recent years, FGDs have also been popularized as a means of qualitative data collection amongst health professionals [13]. The group dynamics of the focus groups elicit deeper and richer data than those obtained from one-to-one interviews [14]. Understanding the specific behaviors of the key actors can be obtained through FGDs and are crucial to identifying risk factors and measuring impact and response. The application of phenomenological research in focus groups can open up new perspectives by gathering information from the different viewpoints of participants [15,16].

The state of Punjab, India, is an agrarian economy, with dairying being an important source of income for farmers. The state is one of the leading milk producers in the country with the highest per capita milk availability [8]. The dairy herds of Punjab face twin challenges of antibiotic overuse and underuse, and lack of antibiotic stewardship [17]. Antibiotic use in the dairy sector of India is predominated by veterinarians (certified veterinary professionals with the basic qualifications of a Bachelor of Veterinary Sciences and Animal Husbandry (B.V.Sc. and AH) degree), para-veterinarians (having veterinary or animal health diploma certificates to assist veterinarians in animal treatment procedures), quacks (persons carrying out treatment practices without any recognized diploma/degree), and the dairy farmer him/herself [8,18]. The predominance of informal practitioners in the health systems of low- and middle-income countries (LMICs), including India, with no formal qualifications, are reported to be the first line of contact for treatment and antibiotic use [19]. Moreover, antibiotic stewardship in India faces huge lacunae due to the over-the-counter access to antibiotics without a medical prescription, and the direct marketing of drugs to the farmers [18,20]. Developing countries are also blamed for the lack of stringent regulations for the use of antibiotics in animals, adherence to withdrawal times, and antibiotic residue testing [21]. For proper antibiotic stewardship in animal health, the roles of animal health service providers and those who sell veterinary drugs are crucial [22,23]. As medical stores/chemist shops are the main sources of antibiotics in the community, chemists play a vital role in the practice of dispensing drugs without prescription, complexed by various factors, such as consumer pressure and economic motives [24]. The positive impact of appropriate education and training on the responsible use of antibiotics and AMR for chemists in developing countries has been highlighted in previous studies [6,25]. Further, dairy quality managers have a vital role in tracing the antibiotic residues in milk at the procurement sites to ensure the availability of antibiotic-free milk to the consumers.

With this background, qualitative research on different key dairy chain stakeholders can elicit a holistic analysis of the intrinsic and extrinsic drivers of antibiotic use and how these factors influence each other to facilitate injudicious antibiotic usage and thereby AMR. Moreover, there are limited systematic studies addressing the prevailing antibiotic usage patterns and resistance in the animal husbandry sector in Indian settings. By investigating antimicrobial use through the dairy chain and how and why they are used, the “hotspots” for priority intervention and communications can be identified.

The present qualitative study employed focus groups and key informant interviews with major stakeholder groups involved in veterinary services in dairy herds of Punjab, such as veterinarians, para-veterinarians, chemists, and dairy quality managers. The discussions focused on the underlying factors regarding AMU and AMR in the dairy chain of Punjab, with a broader objective of enabling the environment for behavioral change and policy intervention for judicious antibiotic usage.

## 2. Materials and Methods

The study was conducted in March 2020 and May 2021. The veterinarians, para-veterinarians, chemists, and dairy quality managers were invited from three districts of Punjab, one from each of the three regions (Majha, Malwa, and Doaba) of the state to participate in FGDs and key informant interviews (KII). The focus groups were conducted with various stakeholders in homogenous groups (i.e., in terms of sharing the same profession) where the participants were free to express their opinions and beliefs about the discussion themes.

### 2.1. Ethics Approval

Individual permission from each participant was obtained for his/her voluntary participation in the study. The Human Ethical Research Committee, Dayanand Medical College and Hospital, Ludhiana, provided the necessary ethical approval to conduct the study under the Indian Council of Agricultural Research, Niche Area of Excellence project on ‘Antibiotic Resistance: Animal Human Interface’ (reference number DMCH/R&D/2021/7).

### 2.2. Study Participants

Four distinct groups, viz. practicing field veterinarians, para-veterinarians, chemists, and dairy quality managers participated in the study. The participants were selected through convenience sampling based on their required experience on the topic and availability. The inclusion criteria were meant to include participants with experience in their respective professions. Large animal field veterinarians (*n* = 63) and para-veterinarians (*n* = 34) above 25 years of age and having job experience in the government sector of over two years were invited. Chemists (*n* = 32) involved in the dairy animal’s pharmacy and dairy quality managers (*n* = 16) from dairy co-operatives and large commercial dairy farms were invited. The FGDs and interviews were conducted in the *Punjabi* language (a regional language of Punjab). A total of 9 homogenous FGDs (6 FGDs of veterinarians, 3 FGDs each of para-veterinarians) were held with 56 veterinarians and 28 para-veterinarians, and the KIIs were held with 18 chemists and 12 dairy quality managers, respectively. The concept of theoretical saturation was used to determine the sample sizes for FGDs and KIIs [26].

The number of participants per focus group ranged from 8 to 12 participants with a median of 10 participants. There were nine ‘dual moderator focus group’ meetings in the study conducted in online mode due to COVID-19 concerns. The discussions were conducted using a semi-structured interview guide with open-ended questions and were facilitated by two moderators (Supplementary Table S1), with a notetaker in the local language.

In brief, the topic guides included relevant discussion points on various arenas for targeted stakeholders as described below:

*For veterinarians:* Antimicrobial usage patterns for common diseases in dairy cattle; various types of antibiotics commonly in use in dairy cattle; completion of antibiotic course duration; alternatives to antimicrobials used; risk practices for antimicrobial resistance; field laboratory support for disease diagnosis and antimicrobial sensitivity testing; awareness of antimicrobial resistance, and suggestions to combat antimicrobial resistance.

*For para-veterinarians:* Self-treatment behaviors among farmers; misuse of antibiotics in the field; peer learning about antibiotic usage; knowledge about antimicrobial resistance, and suggestions to combat antimicrobial resistance.

*For chemists:* Direct marketing of veterinary antibiotics to consumers; frequency of antibiotics sales without prescription; market demand of various antibiotics; awareness of antimicrobial resistance; suggestions for judicious antibiotic prescription and sale.

*For dairy quality managers:* Antibiotic residue testing scenario among dairy farmers; knowledge and perceptions regarding antimicrobial resistance; suggestions for combating antimicrobial resistance in the dairy chain.

Prior to the FGD, the interview guide was pre-tested in two ‘mock’ discussions with the expert academicians of the University. The FGDs lasted for 60–90 min, were recorded, transcribed verbatim, and checked for accuracy. 

The KIIs were held using a semi-structured interview guide following a face-to-face and/or telephone approach by a panel of two researchers, one to ask questions and the other to record answers. Due to COVID-19 concerns and the work pattern of the selected stakeholders, the KIIs were preferred over the FGDs. Moreover, the group dynamic of FGDs may have prohibited these groups of participants from openly discussing sensitive topics, such as AMR and injudicious sales/usage of antibiotics. The interviews took place at the workstations of the individual participants after establishing a suitable rapport and their convenient timings. All interviews lasted for 35–50 min with a median of 43 min and were transcribed verbatim and translated from Punjabi to English.

### 2.3. Data Analysis

The data analysis was guided by the thematic analysis approach, using a bottom-up, inductive approach [27]. In the initial coding, numerous category codes were generated without limiting the number of codes [28]. The coding was done independently by two researchers and then compared and reached a consensus on codes and emerging ideas. The themes were identified from the keywords frequently used by the participants and their emerging ideas were validated by line-by-line analysis across the different transcripts by the first and second authors. From the independent analysis of the transcripts, the relationship ideas were created and categorized into five thematic areas and various subthemes. Each excerpt included code letters (V for veterinarians, PV for para-veterinarians, C for chemists, DQM for dairy quality managers and FGD for the focus group discussion, and KI for key informant), the number of the interview/discussion, and the study setting (R1-Majha, R2-Malwa, and R3-Doaba). The quantitative data were also obtained during the discussion through group consensus. The interpretations of the research data were presented before veterinary public health experts of the university for further comments and validation.

## 3. Results

**Figure 1:**
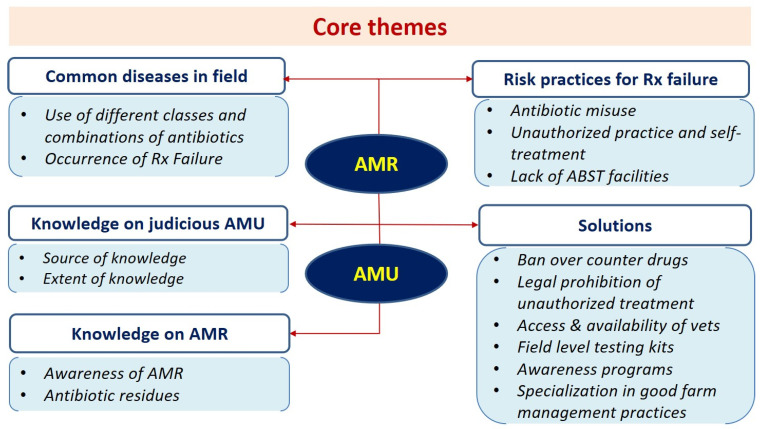
Core themes from focus group discussions and key informant interviews. (Used abbreviations: Rx = veterinary treatment; ABST = antibiotic sensitivity test; AMU = antimicrobial usage; AMR = antimicrobial resistance).

The details of the study demographics of the participants are summarized in Table 1. The analysis of the study generated five core themes and several subthemes and various categories of subthemes, as depicted in Figure 1 and Table 2. The first core theme was the ‘*common disease conditions in the field and their therapy*’. This theme had two sub-themes: (i) use of different classes of antibiotics and antibiotic combinations, and (ii) frequent occurrence of treatment failure in the field. The second core theme was the ‘*risk practices for treatment failure in the field*’ with three sub-themes (i) antibiotic misuse in the field, (ii) unauthorized practitioners (i.e., quackery) and self-treatment by farmers, and (iii) lack of antibiotic susceptibility testing. The third core theme was ‘*knowledge on judicious antibiotic usage*’, which had two subthemes: (i) source of knowledge on antibiotic usage, and (ii) extent of knowledge on antibiotic usage. The fourth core theme was ‘*knowledge on antimicrobial resistance*, with two subthemes: (i) awareness of AMR, and (ii) the antibiotic residues. The fifth core theme was ‘*solutions for curbing the problem*’ with six subthemes: (i) stopping over-the-counter availability of drugs, (ii) legal prohibition of unauthorized treatment, (iii) access and availability of veterinarians, (iv) need for field-level testing kits, (v) need for widespread awareness programs, and (vi) specialization in good managemental practices.

**Theme 1: Common Disease Conditions in the Field and their Therapy**

**Veterinarians:** All the veterinarians agreed that mastitis followed by pyrexia of unknown origin (PUO) and metritis are conditions that require huge antibiotic use in the field. 

V3_FGD1_R1: “In case of pyrexia of unknown origin, without having any clue about the causative agent we are forced to use antibiotics.” “Mixed infections are usually prevalent; metritis or mastitis cases are accompanied by blood parasite infections in most cases”.

**Para-veterinarians:** Regarding diseases/conditions requiring antibiotic usage prevalent in their respective areas, most of the para-veterinarians agreed upon mastitis as the most common infection followed by reproductive problems, fever, tick infections, diarrhea, and skin problems.

PV3_FGD1_R1: “The maximum amount of antibiotics (around 70%) are being used in mastitis cases in my area.”

*Subtheme 1.1: Use of Different Classes of Antibiotics and Antibiotic Combinations*

**Veterinarians:** As per expert estimates from the group, the enrofloxacin and gentamicin combination was the preferred choice of drug for mastitis by 70% of the veterinarians, followed by the third and higher generation of cephalosporins. In the case of metritis, the combination of intrauterine use of metronidazole and parenteral use of fluoroquinolones, such as enrofloxacin or ciprofloxacin, was used by 65% of the doctors, with the rest having a preference for oxytetracycline and ceftriaxone. Around 90% of the veterinarians agreed on using oxytetracycline in case of pyrexia of unknown origin.

V43_FGD5_R3: “The mastitis cases we got to handle are previously mishandled by many hands, we are forced to use higher generation antibiotics or even in many combinations to save the life of the animal. We are left with no other options.”

V15_FGD2_R1: “In repeat breeding or infertility cases also, there is much blind use of higher generation antibiotics, without knowing the actual cause behind that condition.”

**Para-veterinarians:** The widespread use of antibiotic combinations was observed among the para-veterinarians. Overall, the majority of respondents reported the usage of enrofloxacin, a higher generation of cephalosporins, such as ceftriaxone, gentamicin, or oxytetracycline, either alone or in combination.

PV13_FGD2_R2: “Enrofloxacin and gentamicin are the most effective combination for mastitis.”

**Chemists:** The respondents reported the maximum number of sales for quinolones, third- (and higher) generation cephalosporins, tetracyclines, and aminoglycosides as compared to narrow-spectrum drugs, such as penicillin.

C11_KII_R3: “In veterinary drugs, most sales are for drugs such as enrofloxacin, gentamicin, cephalosporins, and oxytetracycline.”

*Subtheme 1.2. Frequent Treatment Failure in the Field*

**Veterinarians:** The frequent occurrence of treatment failure was shared by most veterinarians.

V10_FGD2_R1: “In more than 30% of cases, our first line of treatment fails.” “Many a times treatment failure is observed in mastitis cases, after that I usually have to use the combination of various drugs for effective treatment.”

**Theme 2: Risk Practices for Treatment Failure in the Field**
*Subtheme 2.1. Antibiotic Misuse in the Field*

**Veterinarians:** There was widespread use of antibiotics even in conditions that did not require their usage. Most of the veterinarians pointed toward antibiotic misuse in the field by various veterinary service providers, including para-veterinarians and illegal service providers (i.e., quacks). Many of the para-veterinarians enjoyed the status of doctors among the farmers.

V11_FGD2_R1: “A big problem with antibiotic misuse in the field is that despite our efforts to complete the antibiotic course, as soon as the farmers see the improvement in the health of the animal, they feel reluctant to call us again the next day and to complete the treatment.”

V8_FGD1_R1: “Terramycin (oxytetracycline) is even used as preventive measures by many farmers. Quacks know names of a few antibiotics, terramycin is famous among the quacks.”

V14_FGD2_R1: “Overuse as well as underuse of antibiotics is there in the field. Dose calculation is usually based on 300 kg, but now most cows are cross-bred which weigh above 500 kg and the buffaloes weigh even more.”

**Para-veterinarians**: All of the para-veterinarians, though legally not permitted to treat animals by administering antibiotics, were involved in the treatment of sick animals. Although they knew about bacterial and viral infections, around 90% of the para-veterinarians reported that they gave antibiotics for viral infections. Many of them were unaware of treatment durations, and most of the respondents reported stopping treatment when the animal showed improvement. The participants never had the feeling that they were not entitled to treat animals. They themselves were addressed as doctors by most of the villagers.

PV23_FGD3_R3: “We are no less than veterinary doctors. Veterinary doctors have administrative works more; in fact, we do more treatment than them in villages.”

PV12_FGD2_R2: “We give injections in all cases, without injections there will be no improvement in the animal”. “In indigestion and bloat also, we need injections.”

Subtheme 2.1.1: Incomplete Treatment Duration

**Veterinarians:** The quacks, farmers, and para-veterinarians were blamed by veterinarians for mishandling cases and incomplete treatment regimes.

V6_FGD1_R1: “Quacks, farmers and para-veterinarians give a fixed amount of antibiotics, say 20 mL till the animal show symptoms.”

**Para-veterinarians:** The participants were unaware of any treatment schedule and they stopped treatment once the animal stopped showing symptoms.

PV9_FGD1_R1: “Ceftizoxime is one such drug which gives effect in the animal in a single shot of injection, otherwise we would have to go for two days or till the animal stop showing symptoms.”

Subtheme 2.1.2. Availability of Antibiotics without Prescription

**Veterinarians:** The participating veterinarians observed that as long as medicines are available over the counter in India, antibiotic abuse will continue. They reported that most of the medical shops had untrained employees that supplied medicines over the counter.

V48_FGD6_R3: “The owners of medical shops used to prescribe antibiotics for animals and they even fool the farmers by forcing them to buy the high-end antibiotics to promote various pharmaceutical companies.”

V41_FGD5_R3: “More than 80% of the antibiotic usage in the field is without prescription of a registered veterinarian.”

**Para-veterinarians:** Medical shops and pharmaceutical companies were reported as the main sources of antibiotics for para-veterinarians.

PV18_FGD3_R3: “We learn the treatment from our experience with doctors or our peers; moreover, we used to have good relation with chemists of our area, they used to inform us about the available new drugs.”

PV2_FGD1_R1: “Cefquinome (fourth gen. cephalosporin) is one such drug which is showing promising results in mastitis, I came to know about it from the chemist.”

**Chemists**: All of the participants agreed that they routinely gave antibiotics even without prescriptions. The respondents observed that being involved in business, they could not afford to lose clients if they stopped giving antibiotics without a prescription. They pointed out that due to the lack of laws/regulations in the country, this practice will continue. Around 65% of the respondents agreed that they were unfamiliar with the dosage regime of veterinary antibiotics.

C3_KII_R1: “It’s all about our client, everything is business, we can’t demand a prescription from customers.”

*Subtheme 2.2: Unauthorized Practitioners (i.e., Quackery) and Self-Treatment by Farmers*

**Veterinarians:** The veterinarians observed that the poor farmers mostly resorted to local quacks, and even some of the progressive farmers are involved in self-treatment of the animals. The respondents pointed out the very low ratio of veterinarians per animal in the state as one of the reasons why farmers are forced to conduct self-treatment or call a quack.

V12_FGD2_R1: “Even if I avoid antibiotics, farmers are not satisfied, they will call local quacks or at least purchase bolus of oral antibiotics.”

V37_FGD4_R2: “The prescriptions of quacks are highly shocking, minimum 3 or 4 antibiotics along with some appetite stimulants or anti-inflammatory drugs are given.”

**Para-veterinarians**: Even the para-veterinarians complained about the widespread mishandling of the cases by local quacks, which indicates the widespread prevalence of quackery.

PV12_FGD2_R2: “We used to treat cases in our local area and our cases are complicated by local quacks.”

**Dairy quality managers:** The respondents reported that veterinarians have a minor role in antibiotic usage in the village and that local quacks have a major role in the treatment.

Subtheme 2.2.1. Lack of Availability of Veterinarians

**Veterinarians:** The ratio of veterinarians to the animal population was reported as very low in Punjab.

V16_FGD2_R1: “In Punjab, as per 20th livestock census, there are around 4,000,000 buffaloes and 2,500,000 cattle population; however, there are only 1500–2000 actively working large animal field veterinarians in the State.”

V4_FGD1_R1: “Vets are always busy with administrative work or are overburdened by cases, we hardly find time for health consultancy.”

V25_FGD3_R2: “There is a need to strengthen the veterinary husbandry structure in Punjab. The ratio of veterinarians per animals’ is very low. There is a need to recruit more and more veterinarians to tackle the animal treatment and welfare related issues effectively.”

**Para-veterinarians:** The respondents reported that they are more easily accessible than veterinary doctors in the area.

PV18_FGD2_R2: “We do agree that we get more cases because we are available in the village itself 24 * 7 and our government jobs fetch us more reputation and cases from the village.”

Subtheme 2.2.2. Influence of Pharmaceutical Companies

**Veterinarians:** The respondents observed that the practice of farmers stockpiling antibiotics is common on large farms. They observed that even the progressive farmers who could afford veterinary treatment stocked antibiotics for emergency use and were mostly influenced by the representatives of pharmaceutical companies.

V43_FGD5_R3: “Costly and higher-end antibiotics, such as ceftizoxime and cefquinome are being pushed by the pharma sector. The farmers who are troubled by the frequent recurrence of mastitis usually resort to these drugs, which is influenced by the claims of the pharma companies”.

*Subtheme 2.3: Lack of Antibiotic Susceptibility Testing*

All of the veterinarians agreed that antibiotic susceptibility testing is difficult in field conditions. None of the para-veterinarians in the present study carried out antibiotic sensitivity testing, although around 19% knew that such tests can be used to understand which antibiotic works in the given case.

Subtheme 2.3.1. Lack of Available Facilities

The veterinarians observed that there were limited facilities in rural areas for performing these antibiotic susceptibility tests. They reported that there were no private labs in villages, and veterinary dispensaries were not equipped with such facilities.

V42_FGD5_R3: “Most of us are working in veterinary dispensaries where there are no facilities for testing. Those working in veterinary polyclinics can only have lab facilities for antibiotic susceptibility testing, which are usually 50–60 km far from our hospitals.”

V1_FGD1_R1: “We used to go for antibiotic susceptibility testing only for complicated cases of drug-resistant mastitis.”

Subtheme 2.3.2. Extra Burden on Farmers

The veterinarians opined that antibiotic sensitivity testing facilities should be available at a subsidized rate for farmers.

V10_FGD2_R2: “Most of the farmers are reluctant to pay the charges for testing, as it overburdens them apart from the treatment costs.”

Subtheme 2.3.3. Need for Rapid and Accessible Testing Facilities

The veterinarians noted that, for the testing rates in the field to improve, there should be field-level testing diagnostic kits available that provide rapid and reliable results.

V23_FGD3_R3: “The results of antibiotic sensitivity testing usually come in three days, by the time we are forced to start the therapy.”

**Theme 3: Knowledge of Judicious Antibiotic Usage**
*Subtheme 3.1. Source of Knowledge on Antibiotic Usage*

The most common sources of knowledge for the para-veterinarians were their peers as well as their experiences in assisting veterinarians.

PV22_FGD3_R3: “We learn about which drugs to use in which conditions mostly by our experience in assisting doctors.”

PV14_FGD2_R2: “We consult each other in case of treatment.”

Subtheme 3.1.1. Influence of Social Media

**Veterinarians**: The veterinarians agreed on the marked rise of telephonic/online prescriptions since the COVID-19 pandemic. They expressed concern over the increased reliability of farmers on their social groups for treatment. They also observed a tendency of some progressive farmers to rely on internet sources for treatment.

V7_FGD1_R1: “WhatsApp groups of the farmers are widely used as a discussion platform for treatment by them. This half-knowledge usually turns disastrous and complicates the case.”

V11_FGD2_R1: “Such practice of treatment even through images or video calling should never be entertained”. “After COVID-19, we are forced to attend many of the cases through video calling apps.”

**Para-veterinarians:** Most para-veterinarians who were under 35 years of age reported the influence of social media platforms in their treatments. They observed that even farmers frequently contacted them at odd hours via video calling or by clicking on images.

PV22_FGD3_R3: “We have WhatsApp groups in which we share images and treatment details.”

*Subtheme 3.2. Extent of Knowledge on Antibiotic Usage*

**Para-veterinarians:** More than 70% of para-veterinarians were not able to differentiate between antibiotic and non-antibiotic drugs. None were able to differentiate between broad-spectrum and narrow-spectrum antibiotics. None of the participants were aware of the properties of various antibiotics and their intended actions.

**Chemists:** None of the chemists in the study were formally trained in dispensing veterinary medicines.

C13_KII_R2: “We are aware of human medicines and their dosages, though we keep stock of veterinary medicines also, we are not well versed to advise clients on the intended antibiotic use.”

**Theme 4. Knowledge on Antimicrobial Resistance (AMR)**
*Subtheme 4.1. Awareness of AMR*

**Para-veterinarians:** The participants were unaware of the concept of antimicrobial resistance; around 85% said they experienced some drugs that did not show effectiveness, without a clue as to why.

PV18_FGD2_R2: “Some drugs are not showing results, maybe due to the fraudulent companies who produce these.”

**Chemists:** Many participants were unaware of the concept of antimicrobial resistance.

*Subtheme 4.2. Antibiotic Residues*

**Para-veterinarians:** The para-veterinarians in the study had no idea that humans could be harmed by drinking antibiotic-laden milk. Around 35% of the participants knew antibiotics can be excreted in milk.

PV2_FGD1_R1: “Only drugs for mastitis will be excreted through milk.”

**Dairy quality managers:** Dairy quality managers who participated in the survey were aware of antibiotic residues and their roles in antimicrobial resistance.

DQM11_KII_R3: ‘I have known persons who have developed allergic reactions following consumption of milk of cow treated with strepto-penicillin.”

DQM7_KII_R2: “We know that antibiotic residues may be there in milk. We cannot lose a customer unless there is strict law, we have to continue to accept milk even from treated animals.”

Subtheme 4.2.1. Withdrawal Period

**Veterinarians:** The veterinarians observed that withdrawal periods cannot be observed by farmers as long as farmers are not given suitable compensation for discarding milk.

V36_FGD4_R2: “How can farmers dump the milk without any compensation?” “We can’t even ask a farmer with livelihood dependent on one or two cows to follow withdrawal period.”

**Dairy quality managers:** The respondents agreed that without legal action or compensation, farmers will not observe the withdrawal period.

DQM4_KII_R1: “If the milk societies in an area regularly test and penalize milk sample positive for residues, then only farmers will adhere to the withdrawal period.”

Subtheme 4.2.2. Antibiotic Residue Testing Scenario

**Dairy quality managers:** The residue testing scenario was observed as highly inadequate in field conditions and the cost was pointed out as the main issue.

DQM6_KII_R2: “Nowadays there is the widespread use of antibiotic combinations in the dairy sector, residue detection of various classes requires separate kits/strips and the cost is always the main challenge behind reduced testing of milk samples.”

DQM7_KII_R2: “Only specialized laboratory has chromatography facilities for residue testing, even if we manage to purchase a chromatography machine, its maintenance and functioning are also too costly.”

DQM1_KII_R1: “Many times only in events of conditions such as starter failure, we used to do stringent testing.”

**Theme 5: Solutions for Curbing the Problem**
*Subtheme 5.1. Stopping over the Counter Availability of Drugs*

**Veterinarians:** The veterinarians observed that there should be a legal provision (as in foreign countries) to strictly prohibit antibiotic availability without a prescription.

V19_FGD3_R2: “There is an urgent need for strict enforcement of the law that without veterinary prescription antibiotics should not be available to farmers.”

**Chemists:** The respondents pointed out that unless a stringent law comes into enforcement, the present scenario will continue in India.

*Subtheme 5.2. Legal Prohibition of Unauthorized Treatment or Quackery*

**Veterinarians:** All respondents agreed on the urgent need for legal enforcement against quackery or unauthorized use of antibiotics by unqualified professionals.

V17_FGD2_R1: ‘No quacks are till now punished for their illegal practice.”

*Subtheme 5.3. Access and Availability of Veterinarians*

**Veterinarians:** The respondents insisted on the need to increase the availability of veterinarians–animal ratio in the state. They observed that, unless the availability of veterinary services improves at the farmers’ doorsteps, the illegal treatment will continue.

V24_FGD3_R2: “There are many dispensaries in the state where the veterinarians are not available and have been functioning on the additional charge of nearby vets.”

*Subtheme 5.4. Need for Field-Level Testing Kits*

**Veterinarians:** All of the respondents agreed they were not performing the antibiotic sensitivity tests because of the lack of facilities. The development of rapid field-level kits can be the ideal solution for this.

V14_FGD2_R1: “If there is a provision of reliable and rapid field level kits for antibiotic sensitivity, similar to that of mastitis testing kits in every veterinary dispensary, every doctor will choose an antibiotic based on its results.”

**Dairy quality managers:** The residue testing scenario can be improved in field conditions only by the availability of cost-effective diagnostic kits.

Subtheme 5.4.1. Provision of Compensation to Farmers who Adhere to the Withdrawal Period

**Dairy quality managers:** Antibiotic residues in milk can be reduced only if the farmers are provided with compensation for their losses.

*Subtheme 5.5. Need for Widespread Awareness Programs*

**Veterinarians:** All of the respondents in the present study were aware of the seriousness of the AMR problem and felt the dire need for educating all of society, including consumers, about the risk factors and public health threats of the problem.

V4_FGD1_R1: “There is need to generate awareness among farmers and use of social media should be there to reach to the masses about the dangerous effects of antimicrobial resistance.”

Subtheme 5.5.1. Need for Continuing Veterinary Education Programs

V5_FGD1_R1: “We are not updated on latest policies or latest therapy, there is a huge need for continuing veterinary education programs.”

V18_FGD2_R1: “There is much need for the ‘train the trainer’ program because without upgrading veterinarians we can’t expect the ground level changes.”

*Subtheme 5.6. Specialization in Good Farm Management Practices*

**Veterinarians**: In India, there is a lack of specialization of veterinarians in farm management practices and farm biosecurity aspects.

V13_FGD2_R1: “I strongly believe that mastitis is a managemental problem, many such infectious diseases can be controlled if we make the farmer aware about good farm management practices, for example, we should tell the farmers that mastitis animals should be separated from healthy herd and milked at the end of the herd milking.”

V26_FGD3_R2: “Farm-level biosecurity and management is important to prevent infectious diseases.”

## 4. Discussion

The global action plan on AMR by the World Health Organization (WHO), the Food and Agriculture Organization (FAO), and the World Organization for Animal Health (OIE), along with India’s National Action Plans on AMR, focus on behavioral change-related interventions of the stakeholders through improved awareness and communication. Although India’s National Action Plan on AMR has been enforced to optimize antibiotic use in the country, strict enforcement still needs to be executed at the ground level [9]. Laboratory surveillance on AMR (from different sources) has been widely carried out in India; the scenarios involving antibiotic use and treatment failures remain part of exploratory research. Understanding the specific behavior of the key actor is needed to assess the field-level scenario of antibiotic misuse and determine an efficient solution. Engaging key stakeholders early can help enlist their participation and determine how best to focus interventions on the greatest impacts. The design of interventions based on the surveillance of levels of knowledge and awareness of the stakeholders, which can alter the behaviors of the population and facilitate judicious antibiotic use, is one of the strategic objectives of the global action plan on AMR [29].

In the present study, mastitis was reported as the most common disease condition in dairy cattle requiring antibiotic use by the participating veterinarians and para-veterinarians. This is in accordance with the results of previous studies, where more than 70% of production losses in the dairy sector of India have been attributed to mastitis [18,20,30]. Moreover, there are numerous studies on the isolation of antibiotic-resistant organisms from mastitis milk [31,32,33]. Apart from mastitis, reproductive problems and ‘pyrexia of unknown origin’ were reported as other conditions in which massive antibiotic use has been reported by both veterinarians and para-veterinarians. However, para-veterinarians in the study revealed their practice of administering antibiotics even in non-infectious conditions. The para-veterinarians in the study claimed that they treated more cases than veterinarians in their areas, which is in line with earlier studies that observed that para-veterinarians took much less time than veterinarians in reaching the farmers [34].

The most common antibiotics prescribed by the veterinarians and para-veterinarians in the present study were the highest priority critically important antimicrobials (HPCIA) of the WHO, which mainly included quinolones, such as enrofloxacin, third- and fourth-generation cephalosporins, such as ceftriaxone, cefquinome, etc., high priority critically important antimicrobials, such as gentamicin, highly important antimicrobial, such as oxytetracycline, and aminopenicillins–beta-lactamase inhibitor combinations, such as amoxicillin-clavulanate [18,35]. The present study highlights the pattern of veterinary antibiotic use among animal health workers, which is one of the core recommendations for safeguarding antibiotic effectiveness [36]. A similar pattern of antibiotic usage was reported in earlier studies from India [18] and from Indonesia, where 99.56% of antibiotics used for cattle belonged to veterinary critically important antimicrobial agents (VCIA) [37].

The widespread practice of using antibiotic combinations of HPCIA by both veterinarians and para-veterinarians was reported in the present study. Both groups blamed complications involving the illegal treatment by farmers themselves or ‘quacks’, in which they are forced to use critically important antimicrobials. The veterinarians in the present study reported that they were forced to use broad-spectrum antibiotics since they rarely treat any ‘fresh cases’ untreated by others. In concordance with the present study, most of the animal health workers of Bhutan also reported the practice of using broad-spectrum antibiotics [38].

The present study pointed out the widely prevalent practice of self-treatment behaviors of dairy farmers in the region in accordance with earlier studies from developed nations, such as Canada, where the dairy farmers consider themselves self-reliant to diagnose and treat sick animals with antibiotics [39]; as well as from low- and middle-income countries, such as Kenya and Tanzania [40,41]. Conversely, a recent study from different regions of India pointed out the reliability of farmers on private veterinary practitioners followed by para-veterinarians and veterinarians for treatment [42].

Unlike in earlier studies where most of the para-veterinarians were aware of the concept of AMR [42], the majority of para-veterinarians in the present study were unaware of AMR, although some have observed treatment failures by some of the drugs, which they attributed to fraudulent or sub-standard drugs. The para-veterinarians involved in the present study were unaware of judicious antibiotic use and ideal treatment duration. They reported the usage of antibiotics in a fixed volume until the animal stopped showing symptoms. In line with the present study, where the para-veterinarians could not differentiate between infectious and non-infectious diseases, Sharma et al. [42] also observed that most para-veterinarians administered antibiotics to all sick animals. A similar study on veterinarians and para-veterinarians of Bhutan reported the practice of para-veterinarians using antibiotics for inappropriate durations compared to veterinarians [38].

The present discussion with the dairy quality managers revealed that there was a percentage of farmers who knew that antibiotics could harm themselves, even though they were unaware of how; however, they still sold antibiotic-laden milk to dairies. Similar findings were reported by Patnaik et al. [43] from Punjab—not a single respondent discarded milk from animals treated with antibiotics. According to Sharma et al. [42], a proportion of dairy farmers were aware of the health risks of antibiotic residues but still sold them since they could not afford to waste milk. Some of the earlier studies pointed out that the non-awareness of the farmers regarding the health risks of antibiotic use led to their non-judicious use and non-compliance with the withdrawal periods of drugs [44].

The present animal healthcare infrastructure is grossly inadequate to take care of the vast population of livestock/poultry in the country [45]. The main reason for the quackery and self-treatment behaviors of farmers (pointed out in the present study) was the non-availability of veterinarians. As stated by one veterinarian (V16_FGD2_R1) in the present study, there are around 4,000,000 buffalos and 2,500,000 cattle populations in Punjab that are taken care of by only 1500–2000 actively working large animal field veterinarians. An earlier study by Chauhan et al. [45] observed cost as a major obstacle in seeking professional veterinary services; however, in the present study, even progressive farmers resorted to treatment by unauthorized persons because of the lack of availability of sufficient veterinarians. The limited diagnostic facilities restricted veterinarians from performing even simple tests, such as antibiotic sensitivity, which, even after nine years, is still in accordance with the report of the working group of the Animal Husbandry and Dairying’s 12th five-year plan (2012–2017), concerning limited diagnostic facilities of desired quality in the animal husbandry sector of India [46].

Direct access to veterinary drugs from the chemist’s store without a prescription was reported as the main factor that promoted the misuse of antibiotics by unauthorized persons. Chauhan et al. [45] reported that chemists were the main sources of medicines that farmers used in India. Similar to the result of the present study, earlier studies from Africa reported that antibiotics were mainly sourced at private agro–vet drug shops in the locality [47]. In line with earlier studies, the chemists of medical shops (selling veterinary medicine) who were interviewed in the present study were not trained to advise on veterinary medicine [48].

The veterinarians in the present study urged the need to continue veterinary education programs or regular training for updates on antibiotics and AMR. Similar studies from Bhutan also reported the need for training programs for animal health workers in the country [38]. The lack of proper implementation of antimicrobial stewardship is considered the main driving force of rising AMR in India [49]. Although India has a well-formulated national action plan (for AMR), the progress of the plan is limited due to various hurdles, such as lack of funds, health being an independent affair of corresponding states [50]. Further, the COVID-19 pandemic has shattered the health sector of the country, diverting its resources to prioritize the pandemic response.

## 5. Limitations of the Study

The present qualitative study adopted FGDs and KIIs among various stakeholders of the dairy chain to explore the factors associated with AMU and AMR. It is well known that qualitative research addresses the nature of the phenomena rather than the measure. Thereby, qualitative research does not intend to provide a representative sample of the target population and does not lead to the same kind of inferences as quantitative methods [51]. Further, the sampling for FGDs and KIIs may have been susceptible to selection bias resulting from the purposive sampling method, as the selected participants might have been more informed or held stronger opinions about AMU or AMR than non-participants. However, purposive sampling of participants allowed for the inclusion of different stakeholders with experiences in different sectors and from different geographical areas to represent a more real-life scenario of antimicrobial usage in the dairy sector.

## 6. Conclusions

The present study addresses the context of antibiotic usage in the dairy chain in the state of Punjab. The qualitative study applied a behavioral science lens to identify features that drove the injudicious use of antibiotics in the dairy sector, which could contribute to global, national, and local efforts to curb antimicrobial misuse and resistance. There is a need for the advocation of policies that consider ground-level problems of the dairy sector in Punjab, its challenges, and opportunities, to control AMR, by improving access to veterinary services and improving their prudent use via stringent regulations. There is an urgent need to improve the ratio of veterinarians per animal to strengthen the animal health care system in the region. Moreover, a deeper understanding of the drivers of injudicious antibiotic usage can also guide policy-level decisions in tropical and subtropical regions and countries with similar dairying conditions. This study also paves the way for a participatory approach, linking policymakers and researchers with relevant stakeholders involved, and focusing on need-based priority actions according to each sector and geographic area.

## Figures and Tables

**Table 1:** The demographic characteristics of the study participants.

Variable	Categories	Veterinarians (*n* = 56)	Para-Veterinarians (*n* = 28)	Chemists (*n* = 18)	Dairy Quality Managers (*n* = 12)	Total (%) (*N* = 114)
Sex	Male	39	28	16	12	95 (83.3)
	Female	17	0	02	0	19 (16.7)
Age	20–30	12	8	7	0	27 (23.7)
	31–40	29	7	3	5	44 (38.6)
	41–60	15	13	8	7	43 (37.7)
	>60	0	0	0	0	0
Area	Rural	48	16	12	10	86 (75.4)
	Urban	3	0	6	2	11 (9.6)
	Both	5	12	0	0	17 (14.9)
Education	10th	0	0	0	0	0
	11–12th	0	0	4	0	4 (3.5)
	Diploma	0	28	14	0	42 (36.8)
	Bachelor’s degree	35	0	0	12	47 (41.2)
	Master’s degree	21	0	0	0	21 (18.4)
	PhD degree	0	0	0	0	0
Main occupation	Government	56	28	0	12	96 (84.2)
	Private	0	0	18	0	18 (15.8)
Experience	2–10	12	9	6	7	34 (29.8)
	11–20	31	16	10	5	62 (54.4)
	21–30	11	2	2	0	15 (13.2)
	>30	2	1	0	0	3 (2.6)

**Table 2:** Various discussion themes and subthemes emerged from the focus group discussions (FGDs) and key informant interviews (KIIs) of various stakeholders.

Themes	Subthemes			
	Veterinarians	Para-Veterinarians	Chemists	Dairy Quality Managers
Common disease conditions and their therapy	Use of different classes of antibiotics and antibiotic combinations Frequent treatment failure in field	Use of different classes of antibiotics and antibiotic combinations	Sale of various antibiotics and antibiotic combinations	
Risk factors for treatment failure in the field	Antibiotic misuse in the field Incomplete treatment duration Availability of antibiotics without prescription Unauthorized practitioners and self-treatment by farmers Lack of availability of veterinarians Influence of pharmaceutical companies Lack of antibiotic susceptibility testing	Antibiotic misuse in the field Incomplete treatment duration Availability of antibiotics without prescription Unauthorized practitioners and self-treatment by farmers Lack of availability of veterinarians	Availability of antibiotics without prescription Influence of pharmaceutical companies	Unauthorized practitioners (i.e., quackery) and self-treatment by farmers
Knowledge of judicious antibiotic usage	Source of knowledge on antibiotic usage Influence of social media	Extent of knowledge on antibiotic usage Influence of social media	Extent of knowledge on antibiotic usage	
Knowledge on antimicrobial resistance	Antibiotic residues in animal foods Withdrawal period	Awareness of antimicrobial resistance	Awareness of antimicrobial resistance	Antibiotic residues in animal foods Withdrawal period Antibiotic residue testing
Solutions for curbing the problem	Stopping over-the-counter availability of drugs Legal prohibition of unauthorized treatment Access and availability of veterinarians Need for field-level antibiotic sensitivity and residue testing kits Need for widespread awareness programs Specialization in good farm management practices	Need for widespread awareness programs	Stopping over-the-counter availability of drugs Need for widespread awareness programs	Need for field-level antibiotic sensitivity and residue testing kits Compensation to farmers for following milk withdrawal period Need for widespread awareness programs

## Data Availability

The data presented in this study are available on request from the corresponding author. The data are not publicly available due to privacy and confidentiality agreements to the participants.

## Supplementary Materials

The following supporting information can be downloaded at: https://www.mdpi.com/article/10.3390/antibiotics11091229/s1, Supplementary Table S1—Topic guide for focus group discussion and key informant interview.
