# Supplementary material for: Qualitative Study on Antimicrobial Usage and Resistance in the Dairy Chain: A Situation Analysis and Solutions by Stakeholders from Punjab, India
Source: Antibiotics (Basel). 2022 Sep 9;11(9):1229. doi: 10.3390/antibiotics11091229 (PMC9495224; doi:10.3390/antibiotics11091229)
Supplement: Supplementary file 1 [file antibiotics-11-01229-s001.zip › antibiotics-1858745-supplementary.pdf]

antibiotics

MDPI

Supplementary File

Table S1. Topic guides for focus group discussion and key informant interview.

Focus group interview schedule - Veterinarians

Introduction	Comments
Greet everyone	Make sure everyone has joined the group and comfortable with the internet connections
Introduce everyone in the team	**Moderator:**<br>**Assisted by:**<br>We are going to record the video which will be highly confidential for research purpose only.<br>**Note takers**: _____will note the important points during the discussion
Let the participants introduce themselves	Does everyone know each other?<br>Is the group homogenous in some aspects?
**Introduce the topic for the focus group:**<br>As a research group, we are working on "Antibiotic resistance: Animal-Human Interface". We are interested in Veterinarian's experiences and suggestions as you are the primary stakeholders on this issue.<br>The topic for this focus group is **antibiotic usage pattern and ways to tackle the rising antimicrobial resistance in dairy herds of Punjab**	Explain to the participants that:<br>- There is no right or wrong answer.<br>- You don't have to agree within the group.<br>- Feel free to talk to each other, exchange views, ask questions, and comment on what the other participants say.
**Informed consent**	There will be coding for the participants and names of participants will not be used in the report.<br>All team members agreed to take part voluntarily. If anyone does not want to participate- free to leave the group at any time
**Discussion**	
**Main topic 1**	
**Can you share some most common diseases requiring antibiotic usage in field?**<br>**Common diseases**<br>- Mastitis/Metritis/hemoprotozoan infection/Pyrexia of unknown origin<br>**Antibiotic usage**<br>- Which drugs do you use as the primary line of treatment in various conditions?<br>- Primary line of treatment in Mastitis?<br>- Antibiotic combination in Mastitis?<br>- Antibiotic combination in Metritis?<br>- What is your line of treatment for PUO (Pyrexia of unknown origin)/ recurring fever?<br>- What is your take on usage pattern of antibiotics in bovines? Underuse or Overuse? Why?<br>- Narrow spectrum or broad antibiotics?<br>- Practice of using higher generation antibiotics?<br>- Do you know about HPCIA? In what conditions are HPCIA used in bovines?<br>- What is your take on restricting the use of HPCIA in veterinary sector?<br>- Long-acting drugs? In what conditions?<br>- Alternative systems of medicine- Homeopathy in mastitis?<br>- What are your views on control of mastitis by non-antibiotic alternatives?	
**Main topic 2**	
**What are the main factors which affect your prescribing decisions? (**Why or why not?)	

- Internal factors (Own experience, updates on effective drugs in field)<br>- External factors (cost, farmer pressure, animal)<br>- Use of antibiogram before going to second line of treatment?<br>- How far lab results help in choice of your antibiotics?<br>- Availability of diagnostic labs nearby?<br>- Withdrawal period?	
**Main topic 3**	
**Treatment failure in field**<br>- Which all diseases were difficult to respond to treatment?<br>- If your first line of treatment does not respond, what is your next line of treatment drugs?<br>- How often do you encounter treatment failure in field?<br>- Have you found region specific variation in sensitivity of drugs?<br>- AMR action plans- awareness<br>- Have you received any training on antimicrobial resistance?<br>**Risk practices for AMR in field?**<br>- Role of Quacks? What are their risk practices?<br>- Role of para-vets?<br>- Role of Farmers? Risk practices?<br>- Role of Vets?<br>- Vocal prescription<br>- Prescription through social media like WhatsApp<br>- Withdrawal period?<br>- Stock pile of antibiotics on farm?<br>- Antibiotic stewardship<br>- Availability over the counter?	-
**Main topic 4**	
**Can you give suggestions on how to tackle the rising AMR?**<br>- Suggestions at field level<br>- **Vaccination at field level?** How many diseases are covered in vaccination policy of government?<br>- **Any other available vaccines which are not covered in Govt vaccination policy?**<br>- Changes in management/Biosecurity practices<br>- Suggestions at research level<br>- Suggestions at policy level<br>- Have you previously tried to do this?<br>- Do you know someone else who tried this? (What happened?)	
**Wrap-up**	
- Summarize the discussion	Summarize main topics to reach a consensus
**Thank participants**<br>- Does anyone have any questions or other suggestions?<br>- Repeat confidentiality from introduction, all participants are OK with this?	Suggest we finish discussion<br>Make sure contact details to participants are saved<br>Make sure participants leave feeling comfortable!

Focus group interview schedule: Para-Veterinarians

Introduction	Comments
Greet everyone	Make sure everyone has joined the group and comfortable with the internet connections
Introduce everyone in the team	**Facilitator:**<br>**Assisted by:**

	We are going to record the video which will be highly confidential for research purpose only.<br>**Note takers:**
**Introduce the topic for the focus group:**<br>As a research group, we are working on "Antibiotic resistance: Animal-Human Interface". We are interested in para-veterinarian's experiences and suggestions as you are the primary stakeholders on this issue.<br>The topic for this focus group is **antibiotic usage pattern and ways to tackle the rising antimicrobial resistance in dairy herds of Punjab**	Explain to the participants that:<br>- There is no right or wrong answer.<br>- You don't have to agree within the group.<br>- Feel free to talk to each other, exchange views, ask questions, and comment on what the other participants say.
**Informed consent**	There will be coding for the participants and names of participants will not be used in the report.<br>All team members agreed to take part voluntarily. If anyone does not want to participate- free to leave the focus group at any time
**Discussion**	
**Main topic 1**	
**Can you share some most common diseases in your farm and the treatment strategy?**<br>**Common diseases**<br>- Mastitis/Metritis/hemoprotozoan infection/Pyrexia of unknown origin<br><br>**Antibiotic usage**<br>- Which drugs do you use as the primary line of treatment in various conditions?<br>- Primary line of treatment in Mastitis?<br>- Antibiotic combination in Mastitis?<br>- Metritis?<br>- Antibiotic combination in Metritis?<br>- What is your line of treatment for PUO (Pyrexia of unknown origin)/ recurring fever?<br>- Long-acting drugs? In what conditions?<br>- Availability over the counter?<br>- Alternative systems of medicine- Homeopathy in mastitis?	NOTE! For all topics try to have participants talk about their **experiences**, rather than opinions. Let participants **tell their story**, ask prompting questions only if needed.
**Main topic 2**	
**What are the main barriers in following proper treatment schedule?**	• How many of you follow three days/five-day treatment course?<br>• Once the symptoms are relieved, do you stop treatment?<br>• Are the antibiotics widely available over the counter?
**Main topic 3**	
**Treatment failure in field**<br>- Which all diseases were difficult to respond to treatment?<br>- How often do you encounter treatment failure in field?	• Out of 10 cases of mastitis, how many fail to respond to your first line of treatment?<br>• Out of 10 cases of metritis, how many fail to respond to your first line of treatment?
**Risk practices for AMR in field?**<br>Advice on Withdrawal period	• How many of you know antibiotics appear in the milk of treated animals?<br>• Are there any health hazards associated with consumption of milk containing antibiotics?
**Main topic 4**	
• What are your views on control of mastitis by **non-antibiotic alternatives**?<br>• How to do you stay updated about treatment practices?	

Wrap-up	
• Summarize the discussion	Summarize main topics to reach a consensus
**Thank participants**<br>- Does anyone have any questions or other suggestions?<br>- Repeat confidentiality from introduction, all participants are OK with this?	Suggest we finish discussion<br>Make sure contact details to participants are saved<br>Make sure participants leave feeling comfortable!

Focus group interview schedule: Dairy Farmers

Introduction	Comments
Greet everyone	Make sure everyone has joined the group and comfortable with the internet connections
Introduce everyone in the team	**Moderator:**<br>**Assisted by:**<br>We are going to record the video which will be highly confidential for research purpose only.<br>**Note taker:**
Let the participants introduce themselves	Does everyone know each other?<br>Is the group homogenous in some aspects?
**Introduce the topic for the focus group:**<br>As a research group, we are working on "Antibiotic resistance: Animal-Human Interface". We are interested in Dairy Farmer's experiences and suggestions as you are the primary stakeholders on this issue.<br>The topic for this focus group is **antibiotic usage pattern and ways to tackle the rising antimicrobial resistance in dairy herds of Punjab**	Explain to the participants that:<br>- There is no right or wrong answer.<br>- You don't have to agree within the group.<br>- Feel free to talk to each other, exchange views, ask questions, and comment on what the other participants say.
**Informed consent**	There will be coding for the participants and names of participants will not be used in the report.<br>All team members agreed to take part voluntarily. If anyone does not want to participate- free to leave the focus group at any time
**Discussion**	
**Main topic 1**	
**Can you share some most common diseases in your farm and the treatment strategy?**<br>**Common diseases**<br>- Mastitis/Metritis/hemoprotozoan infection/Pyrexia of unknown origin<br><br>**Antibiotic usage**<br>- Which drugs do you use as the primary line of treatment in various conditions?<br>- Line of treatment in Mastitis?<br>- Antibiotic combination in Mastitis?<br>- Metritis?<br>- Antibiotic combination in Metritis?<br>- What is your line of treatment for PUO (Pyrexia of unknown origin)/ recurring fever?<br>- Practice of using higher generation antibiotics?<br>- Long-acting drugs? In what conditions?<br>- Stock pile of antibiotics on farm?<br>- Availability over the counter?<br>- Alternative systems of medicine- Homeopathy in mastitis?	NOTE! For all topics try to have participants talk about their **experiences**, rather than opinions. Let participants **tell their story**, ask prompting questions only if needed.<br><br>• What % of farmers stockpile antibiotics in field?<br>• Which all antibiotics you store in your farm?<br>• Are the antibiotics widely available over the counter?
**Main topic 2**	

**What are the main barriers in following proper treatment schedule?**<br>Non availability of vets?<br>Expensive treatment by vets?<br>Self-knowledge of treatment?<br>Reoccurrence of diseases once treated by vets	• How many of you follow three day/five day treatment course?<br>• Once the symptoms are relieved, do you stop treatment?<br>• How many of you do not have access to veterinarian?
**Main topic 3**	
**Treatment failure in field**<br>- Which all diseases were difficult to respond to treatment?<br>- How often do you encounter treatment failure in field?<br><br>**Risk practices for AMR in field?**<br>- **Vocal prescription**<br>- **Prescription through social media like WhatsApp**<br>- **Withdrawal period?**	• In how much % of mastitis cases, do farmers treat themselves?<br>• In how much % of mastitis cases, do farmers call vets?<br>• In how much % mastitis cases, do farmers call private doctors?<br>• In how much % of reproductive problems, do farmers treat themselves?<br>• In how much % of reproductive problems, do farmers call vets?<br>• In how much % reproductive problems, do farmers call private doctors?<br>• Do doctors prescribe antibiotics over phone?<br>• Do you contact doctors through phone/social media?<br>• How many of you sell milk from antibiotic treated cows?<br>• How many of you discard milk from treated cows?<br>• How many of you know antibiotics appear in the milk of treated animals?<br>• Are there any health hazards associated with consumption of milk containing antibiotics?<br>• What % of doctors advise you about withdrawal period?<br>• Are there any residue testing facilities in your area?
**Main topic 4**	
- **Vaccination at field level?** How many diseases are covered in vaccination policy of government?<br>- What are your views on control of mastitis by **non-antibiotic alternatives**?<br>- **How to do you stay updated about dairying? Peer Farmer's?**	• What % of farmers vaccinate their animals in field?<br>• How many of you feel that mastitis can be controlled by managemental factors?<br>• Dry cow therapy? Insertion of tubes?<br>• Teat Dips?<br>• Allowing cow to stand for 30 minutes after milking?<br>• How many of you are members of farmer's association?<br>• How many of you have received any formal training in dairying?<br>• Any social media groups for dairying?
**Wrap-up**	
- Summarize the discussion	• Summarize main topics to reach a consensus
**Thank participants**<br>- Does anyone have any questions or other suggestions?<br>- Repeat confidentiality from introduction, all participants are OK with this?	• Suggest we finish discussion<br>• Make sure contact details to participants are saved<br>• Make sure participants leave feeling comfortable!

Topic Guide for Key Informant Interviews on Antimicrobial Resistance (AMR)

A. CHEMISTS

1. Direct marketing of vet antibiotics to consumers
2. How frequently antibiotics are sold without prescription?
3. Market Demand of various antibiotics
4. Knowledge on veterinary antibiotics
5. Knowledge on AMR
6. Have you been involved in any campaign to raise awareness on AMR?
7. Suggestions for judicious antibiotic prescription

B. DAIRY QUALITY CONTROL MANAGERS

1. Knowledge and perceptions regarding AMR
2. Knowledge and perceptions regarding antibiotic usage in field conditions of Punjab
3. Antibiotic residue testing scenario
4. Suggestions for reducing residues in dairy products
